# Relationship between Circulating PCSK9 and Markers of Subclinical Atherosclerosis—The IMPROVE Study

**DOI:** 10.3390/biomedicines9070841

**Published:** 2021-07-19

**Authors:** Daniela Coggi, Beatrice Frigerio, Alice Bonomi, Massimiliano Ruscica, Nicola Ferri, Daniela Sansaro, Alessio Ravani, Palma Ferrante, Manuela Damigella, Fabrizio Veglia, Nicolò Capra, Maria Giovanna Lupo, Chiara Macchi, Kai Savonen, Angela Silveira, Sudhir Kurl, Philippe Giral, Matteo Pirro, Rona Juliette Strawbridge, Bruna Gigante, Andries Jan Smit, Elena Tremoli, Mauro Amato, Damiano Baldassarre, on behalf of the IMPROVE Study Group

**Affiliations:** 1 Centro Cardiologico Monzino, IRCCS, 20138 Milan, Italy; daniela.coggi@ccfm.it (D.C.); beatrice.frigerio@ccfm.it (B.F.); alice.bonomi@ccfm.it (A.B.); daniela.sansaro@ccfm.it (D.S.); alessio.ravani@ccfm.it (A.R.); palma.ferrante@ccfm.it (P.F.); manuela.damigella@ccfm.it (M.D.); fabrizio.veglia@ccfm.it (F.V.); nicolo.capra@ccfm.it (N.C.); mauro.amato@ccfm.it (M.A.); 2 Dipartimento di Scienze Farmacologiche e Biomolecolari, Università degli Studi di Milano, 20133 Milan, Italy; massimiliano.ruscica@unimi.it (M.R.); chiara.macchi@unimi.it (C.M.); 3 Dipartimento di Scienze del Farmaco, Università degli Studi di Padova, 35122 Padova, Italy; nicola.ferri@unipd.it (N.F.); mariagiovanna.lupo@gmail.com (M.G.L.); 4 Foundation for Research in Health Exercise and Nutrition, Kuopio Research Institute of Exercise Medicine, 70100 Kuopio, Finland; kai.savonen@uef.fi; 5 Department of Clinical Physiology and Nuclear Medicine, Kuopio University Hospital, 70210 Kuopio, Finland; 6 Department of Medicine Solna, Division of Cardiovascular Medicine, Karolinska Institutet, 17177 Stockholm, Sweden; angela.silveira@ki.se (A.S.); rona.strawbridge@glasgow.ac.uk (R.J.S.); bruna.gigante@ki.se (B.G.); 7 Karolinska University Hospital, Solna, 17177 Stockholm, Sweden; 8 Kuopio Campus, Institute of Public Health and Clinical Nutrition, University of Eastern Finland, 70210 Kuopio, Finland; sudhir.kurl@uef.fi; 9 Assistance Publique-Hopitaux de Paris, Service Endocrinologie-Metabolisme, Groupe Hôspitalier Pitie-Salpetriere, Unités de Prévention Cardiovasculaire, 75651 Paris, France; philippe.giral@aphp.fr; 10 Internal Medicine, Angiology and Arteriosclerosis Diseases, Department of Medicine and Surgery, University of Perugia, 06129 Perugia, Italy; matteo.pirro@unipg.it; 11 Institute of Health and Wellbeing, University of Glasgow, Glasgow G12 8RZ, UK; 12 Health Data Research, Glasgow G12 8RZ, UK; 13 Department of Medicine, University Medical Center Groningen, Groningen and Isala Clinics Zwolle, 9700 RB Groningen, The Netherlands; a.j.smit@umcg.nl; 14 Maria Cecilia Hospital, 48033 Cotignola, Italy; etremoli@gvmnet.it; 15 Department of Medical Biotechnology and Translational Medicine, Università degli Studi di Milano, 20129 Milan, Italy

**Keywords:** PCSK9, subclinical atherosclerosis, carotid artery, intima-media thickness, echolucency

## Abstract

(1) Background and purpose: circulating proprotein convertase subtilisin/kexin type 9 (PCSK9) is one of the key regulators of cholesterol metabolism. Despite this, its role as a player in atherosclerosis development is still matter of debate. Here, we investigated the relationships between this protein and several markers of subclinical atherosclerosis. (2) Methods: the IMPROVE study enrolled 3703 European subjects (54–79 years; 48% men; with ≥3 vascular risk factors), asymptomatic for cardiovascular diseases. PCSK9 levels were measured by ELISA. B-mode ultrasound was used to measure markers of carotid subclinical atherosclerosis. (3) Results: in the crude analysis, PCSK9 levels were associated with several baseline measures of carotid intima-media thickness (cIMT) (all *p* < 0.0001); with cIMT change over time (Fastest-IMTmax-progr) (*p* = 0.01); with inter-adventitia common carotid artery diameter (ICCAD) (*p* < 0.0001); and with the echolucency (Grey Scale Median; GSM) of both carotid plaque and plaque-free common carotid IMT (both *p* < 0.0001). However, after adjustment for age, sex, latitude, and pharmacological treatment, all the afore-mentioned correlations were no longer statistically significant. The lack of correlation was also observed after stratification for sex, latitude, and pharmacological treatments. (4) Conclusions: in subjects who are asymptomatic for cardiovascular diseases, PCSK9 plasma levels do not correlate with vascular damage and/or subclinical atherosclerosis of extracranial carotid arteries.

## 1. Introduction

Proprotein convertase subtilisin/kexin type 9 (PCSK9) is a circulating enzyme involved in cholesterol homeostasis. By modulating the expression of low-density lipoprotein receptor (LDL-R), PCSK9 is a key regulator of cholesterol metabolism [1,2] and a potential player in atherosclerosis [3,4] and cardiovascular diseases [5]. The use of transgenic animal models has provided evidence that, in addition to the LDL-R, PCSK9 may also degrade other receptors potentially involved in pro-atherosclerotic processes, including endothelial dysfunction, hemostasis and inflammation [6]. This, combined with the fact that (a) carriers of loss of function variants for PCSK9 show low levels of LDL-cholesterol with a reduction in the risk of coronary events [7], and (b) PCSK9 is expressed in endothelial cells, vascular smooth muscle cells and macrophages [6] suggests that PCSK9 may play a role also in plaque development. However, despite this evidence, conflicting results emerge when the association is investigated on the basis of relationships between circulating levels of this proprotein and the incidence of cardiovascular events. In the literature, indeed, there are studies both supporting [5] and rejecting [8,9,10] the existence of such a role. Besides these relationships, other studies have also highlighted significant associations between PCSK9 and markers of vascular damage, including a positive association with 10-year atherosclerosis progression [11], pulse wave velocity [12], extent of coronary artery calcification [13], and volume of coronary plaques necrotic core [14]. Regarding traditional indices of subclinical atherosclerosis, such as carotid intima-media thickness (cIMT) and plaque-size, both positive [15,16,17,18,19] and null associations [9,20,21,22] were observed.

To add further insights on this issue, in this study we have investigated the relationship between circulating levels of PCSK9 and a series of ultrasonographic indices of vascular damage and subclinical atherosclerosis in a large sample of European subjects.

## 2. Materials and Methods

This work was performed taking advantage of databases, biobanks and an imaging-bank of the IMPROVE study, a European, multicenter, longitudinal, and observational study specifically designed to identify novel biomarkers of subclinical and clinical atherosclerosis [23]. The study included 3703 subjects [54–79 years, 48% men, with ≥3 vascular risk factors (VRFs)], asymptomatic for cardio- and cerebrovascular diseases at the time of recruitment. Participants were enrolled in seven centers of five European countries: Finland (two centers), Sweden, the Netherlands, France, and Italy (two centers) and followed-up for 36 months. Variables collected included clinical, biochemical, genetic, socioeconomic, psychological, nutritional, and educational data, personal and family history of diseases, drug intake, and physical activity. Eligibility criteria for enrollment, objectives, methods, and participants’ baseline characteristics have been previously described [23].

The study complies with the rules of Good Clinical Practice and with the ethical principles established in the Helsinki Declaration and was approved by local Ethics Committees in each study center. All subjects gave written informed consent.

### 2.1. Blood Sampling and PCSK9 ELISA

Blood samples were collected after an overnight fast upon enrolment in the study. Plasma PCSK9 levels were measured in blood aliquots using a commercial ELISA kit (R&D Systems, Minneapolis, MN, USA) able to identify both free and LDL-R-bound PCSK9. Samples were diluted 1:20 in accordance with the manufacturer’s instructions, and incubated onto a microplate, which was pre-coated with a specific monoclonal antibody for the human PCSK9. A four-parameter logistic curve-fit was used to obtain sample concentrations (minimum detectable concentration: 0.219 ng/mL). The intra- and inter-assay coefficients of variability (CVs) were 5.4 ± 1.2% and 4.8 ± 1.0%, respectively.

### 2.2. Measurement of Ultrasonographic Variables

#### 2.2.1. Carotid Intima-Media Thickness (cIMT), Carotid Plaques and Their Changes over Time

The IMPROVE study ultrasonographic protocol has been previously described [23]. Briefly, the far walls of left and right common carotids (CCs), bifurcations (Bifs), and internal carotid arteries (ICAs) were visualized in three different angles (lateral, anterior and posterior). Images were recorded and measurements were made off-line using a specific software (M’Ath, Metris SRL, Paris, France) [24]. Areas with plaques (defined as a maximum cIMT > 1.5 mm) were not excluded from the cIMT measurements.

The changes over time of cIMT and plaque-size were measured as previously described [25]. Briefly, ultrasonographic measurements were repeated after 15 months with the same ultrasonographic protocol used at baseline. The cIMT and plaque-size changes, expressed in mm/year, were calculated as the difference between the 15-month measurement and the corresponding value measured at baseline, divided by the length of the intervening time period. Of course, when 15-months progression variables were considered, the post-progression median follow-up was of about 21 months.

The variables used for the statistical analyses were: IMTmean (i.e., average of IMTmean measured in 1st cm of CC, CC, Bif, and ICA of left and right carotid arteries), IMTmax (i.e., the highest value of IMTmax out of 1st cm of CC, CC, Bif, and ICA of left and right carotid arteries), IMTmean-max [i.e., the average of maximal IMT measured in eight carotid segments (1st cm of CC, CC, Bif, and ICA) in left and right carotid arteries], plaque-free (PF) CC-IMTmean (i.e., IMTmean of the 2nd cm of CCs measured in plaque-free areas), Fastest-IMTmax-progr (i.e., the greatest value chosen among the 15-month progressions of IMTmax detected in the whole carotid tree irrespective of location) and inter-adventitia common carotid artery diameter (ICCAD) (i.e., the average of the inter-adventitia diameter measurements carried out in plaque-free areas of the 2nd cm of left and right common carotid arteries). Results of intra- and inter-assay variability tests were previously described [23,25,26].

#### 2.2.2. Echolucency of cIMT and Echolucency of Carotid Plaques

The quantitative evaluation of echolucency is based on the analysis of the grey scale median (GSM) [27,28,29,30] of pixel distribution, normalized for the echolucency of blood (e.g., zero for maximum black) and for the echolucency of adventitia (e.g., 255 for maximum white). Once defined, such parameters were applied to the entire sequence of frames. To measure carotid echolucency, a series of 120–200 frames of the recorded scans were imported into a dedicated software (MIA Carotid Analyzer, Coralville, IA, USA), able to provide a densitometric analysis of images. A region of interest was manually placed around the segment to be analyzed (i.e., the cIMT or the plaque) and the software automatically calculated the GSM in all the selected frames (three frames for each measurement).

**Figure 1:**
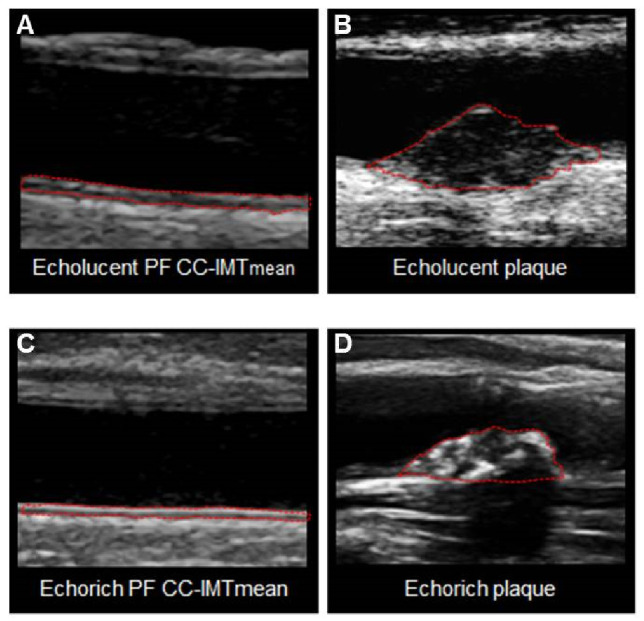
Examples of lesions with echolucent (**A**,**B**) and echo-rich (**C**,**D**) features of two types of carotid ultrasonographic images. Panels on the left show the echolucency features detected on the common carotid IMT, measured in plaque free areas (echolucency of PF CC-IMTmean). Panels on the right show the same features detected on a carotid plaque (echolucency of plaque).

Low GSM values represent an echolucent (dark) cIMT or plaque (Figure 1A,B), whereas high GSM values represent an echo-rich (bright) cIMT or plaque (Figure 1C,D).

For each ultrasonographic scan, the GSM of PF CC-IMTmean was detected in lateral projection, and the GSM of the biggest plaque was detected in the whole carotid tree considering the three scan angles. Both were measured on the far walls of both left and right carotid arteries.

The variables used for the statistical analyses were: the average GSM of left and right PF CC-IMTmean, and the average GSM of the blackest plaque between left and right biggest carotid plaques.

The reproducibility of echolucency measurements was determined in 158 subjects who underwent a first scan at the baseline visit and a second scan two weeks apart. The analysis was performed considering left and right carotid arteries separately. For PF CC-IMTmean, a significant correlation was observed between GSM values of basal and replicate scans (left carotid: CV = 4.95, r = 0.98; right carotid: CV = 5.09, r = 0.95). Bland-Altman plots (data not shown) revealed that the bias between the two measurements was not significant (left carotid: absolute difference = 2.88 ± 2.34; right carotid: absolute difference = 2.81 ± 2.22). Similar results were obtained for carotid plaque echolucency (left carotid: CV = 6.10, r = 0.96, absolute difference = 3.04 ± 2.29; right carotid: CV = 5.98, r = 0.96, absolute difference = 2.84 ± 2.4).

### 2.3. Statistical Analysis

All quantitative variables were reported as mean ± SD. Variables with a skewed distribution were presented as median and interquartile range, and log-transformed before analyses. Categorical variables were reported as frequency and percentage. Trends across PCSK9 quintiles for continuous and categorical variables were assessed by ANCOVA and by Mantel–Haenszel χ^2^-test, respectively.

Multiple linear regression analysis was used to assess the independent relationship between PCSK9 levels and each ultrasonographic variable. Model 0 was unadjusted; Model 1 was adjusted for age, sex, latitude, and pharmacological treatment (i.e., therapy with statins, or fibrates, or with both statins and fibrates). The repeatability of GSM measurements was assessed by using the Bland–Altman approach [31]. All the analyses were carried out with SAS statistical package v. 9.4 (SAS Institute Inc., Cary, NC, USA).

## 3. Results

Plasma PCSK9 levels were successfully measured in 3673 (52.1% women and 47.9% men) out of the 3703 participants included in the IMPROVE study [32]. The baseline characteristics of the study population are reported according to PCSK9 quintiles (Table 1).

A negative trend across PCSK9 quintiles was observed for some continuous variables (i.e., age, diastolic blood pressure, systolic blood pressure, body mass index, waist/hip ratio, educational level, uric acid, blood glucose, creatinine, and estimated glomerular filtration rate) and some categorical variables (i.e., male sex, personal histories of low high-density lipoproteins (HDL)-cholesterol, of hypertension and of diabetes) (Table 1). Conversely, positive trends were found among PCSK9 quintiles and total, LDL-, HDL-cholesterol, and triglycerides (among the continuous variables), as well as personal histories of hypercholesterolemia and hypertriglyceridemia (among the categorical variables) (Table 1). Additional unadjusted relationships among PCSK9 and a series of variables (anthropometric, biochemical, nutritional etc.) are reported in Supplementary Table S1.

The ultrasonographic variables, stratified by quintiles of PCSK9, are shown in Table 2. The trend of association with PCSK9 quintiles was negative with cIMT variables and ICCAD and positive with the GSM of PF CC-IMTmean and GSM of carotid plaque.

Table 3 shows the unadjusted (Model 0) and adjusted (Model 1) relationships between PCSK9 and each ultrasonographic variable considered. In Model 0, PCSK9 was associated with almost all of the variables. All of these associations, however, were no longer significant in Model 1 (after adjustment for age, sex, latitude, and therapies with statins, with fibrates or both statins and fibrates). Almost identical results were observed when Model 1 was stratified by sex (Supplementary Figure S1), by latitude (Supplementary Figure S2), or by pharmacological treatment (Supplementary Figure S3).

## 4. Discussion

The present study shows that, in the IMPROVE cohort, plasma levels of PCSK9 were not associated with any of the carotid ultrasonographic variables considered. Indeed, although in the crude analyses all the ultrasonographic variables appeared to be associated with PCSK9 (Table 2 and Table 3, Model 0), the same associations lost their statistical significance when the analyses were adjusted for confounders, such as age, sex, latitude, and therapies with statins, fibrates, or both (Table 3, Model 1). The relationship between PCSK9 and cIMT has been one of the most investigated [9,15,16,17,18,19,20,21,22]. PSCK9 has been positively associated with cIMT, independently of traditional VRFs, in relatively small studies carried out in specific cohorts, including healthy individuals (*n* = 120) [15], newly diagnosed type 2 diabetes mellitus participants (*n* = 100) [16], systemic sclerosis patients (*n* = 73) [17], hypertensive subjects (*n* = 126) [18], and first-degree relatives of patients with familial hypercholesterolemia who were negative for the familial LDL-R or APOB mutation (*n* = 112) [19]. Conversely, no associations were found in similar analyses carried out in asymptomatic individuals (*n* = 295) [20], in patients with systemic lupus erythematosus (*n* = 109) [21] or with axial spondylarthritis (*n* = 299) [22]. To the best of our knowledge, the only study that has evaluated the association between PCSK9 and cIMT in a relatively large sample was the FATE (Firefighters and Their Endothelium) [9], carried out in 1527 middle-aged men in primary prevention. In line with our findings, FATE also found a correlation between PCSK9 and VRFs, but failed to confirm any correlation with subclinical atherosclerosis observed in the small studies quoted above. It is worth mentioning that the ultrasonographic variables used in the afore-mentioned studies [9,15,16,17,18,19,20,21,22] were the average value of common carotid artery IMT (CC-IMTmean), and/or the maximal IMT value detected in the whole carotid tree. In our study, we measured the IMT in all the segments of the whole left and right carotid trees, thus producing IMT results not only for common carotids, but also for bifurcations and internal carotid arteries, i.e., for those segments where the atherosclerotic process is usually much more prevalent. In addition, by incorporating the plaque in the IMT measurements we have produced variables that are much more strongly related to the atherosclerotic processes than IMT of common carotid arteries measured only in plaque-free areas.

Regarding the cIMT change over time, our results showed that the correlation with PCSK9 observed in the crude analysis is also likely spurious; indeed, it disappears in multivariable analysis. To the best of our knowledge, only one study carried out in 643 subjects in primary prevention has evaluated the relationship between PCSK9 and the change over time of subclinical atherosclerosis [11]. In that study, the indices of subclinical atherosclerosis progression used were the 10-year carotid plaque formation and the 10-year change in Total Plaque Area (TPA). That study highlighted a significant positive association between PCSK9 and the 10-year carotid plaque formation, with a Relative Risk of 1.09 (95% CI: 1.03, 1.15; *p* = 0.003) per increase of one PCSK9 quartile, after adjustment for several confounders. The same authors also investigated, with multivariate analysis, the increase in TPA according to levels of baseline LDL-cholesterol and PCSK9. In such analysis TPA increased with the rising of the baseline levels of LDL-cholesterol (*p* < 0.001) and PCSK9 (*p* = 0.008). It is worth noting that in that study atherosclerosis progression was indexed by the formation of new carotid plaques or by the change in TPA, and not with change in cIMT. This, combined with their much longer (10 years) follow-up, compared to the post-progression one used in our study (21 months), might explain the difference observed with respect to our results. However, in our experience, the use of plaque prevalence instead of change in mean or maximal cIMT does not result in higher predictive power of vascular events [26]; moreover, considering the difference in sample size, even with longer follow-up they accounted for approximately 6800 person-years vs. 6336 in our study.

ICCAD enlargement is a compensatory arterial response occurring in presence of atherosclerotic stimuli including the simple presence of atherosclerosis risk factors [33]. Moreover, the ICCAD enlargement associates with subclinical atherosclerosis [34,35] and, like cIMT, improves risk stratification in asymptomatic individuals [26]. Thus, combined with the positive associations between PCSK9 and atherosclerosis [3,4] or cardiovascular diseases [5], a positive relationship between PCSK9 and ICCAD was expected. By contrast, in our study we found a negative correlation in the crude analysis (Table 2 and Table 3, Model 0) that disappeared after adjustment for possible confounders (Table 3, Model 1). These results suggest a lack of any role for circulating PCSK9 in the compensatory arterial response to VRFs (i.e., ICCAD enlargement). Whether these are reliable or not is difficult to discuss, as to date, there are no published studies on this phenotype that may allow even the simplest comparison. However, the fact that the association disappeared after adjustment for few covariates suggests that the correlation observed in the unadjusted analysis is actually a spurious relationship.

Regarding echolucency, to the best of our knowledge, this is the first study that has investigated the relationship of PCSK9 with echolucency (GSM) of both cIMT and carotid plaque. As most of the studies published so far have shown that GSM is negatively associated with VRFs [36,37,38], and since it is well known that echolucent plaques are those most vulnerable to rupture [30,39,40,41,42], a negative relationship was expected. However, as for cIMT, IMT change over time and ICCAD, we found no association between echolucency and circulating PCSK9 in the multivariable analysis (Table 3, Model 1). This suggests that the weak correlation observed in the unadjusted analyses was likely spurious. The association between PCSK9 and arterial lesion echolucency has been investigated in only one other study [21]. In line with our results, that study did not find any significant association. However, it is worth mentioning that, in that study, the authors focused on plaque echolucency. As we have investigated not only plaque echolucency but also the echolucency of cIMT, our study adds another piece of information to this rarely investigated issue.

### Strengths and Limitations of the Study

The present study has several strengths: first, it analyzes the largest sample so far available investigating the association of PCSK9 with markers of subclinical atherosclerosis; second, it has been carried out in five European countries, thus increasing the results’ generalizability. Furthermore, the acquisition method of carotid images, with: (a) an ultrasonographic scan protocol fully standardized across the seven involved centers, (b) all the operators similarly trained and certified, (c) all scans blindly analyzed in a single reading center and (d) a large number of cIMT variables examined, has provided to the study the potential to identify the most informative carotid segment and/or the best cIMT summary variable to be used. The present study also includes three limitations. First, the use of a simple commercial ELISA method for the assessment of PCSK9 levels, instead of the mass spectrometry that is much more precise but also much more complex. However, the relationships found with VRFs, which are in line with those reported by other authors, testify that our data are reliable. Second, the commercial ELISA assay used does not allow to discriminate between the free form of circulating PCSK9 and the one bound to LDL [43] or to Lp(a) [44] and HDL [45]. Third, the determination of PCSK9 levels was performed only at the beginning of the study without a second assessment at follow-up.

## 5. Conclusions

IMPROVE, which is the largest study evaluating the role of PCSK9 in subclinical atherosclerosis conducted to date, failed to demonstrate any association between circulating levels of PCSK9 and various markers of vascular damage or subclinical atherosclerosis.

Our results do not support the hypothesis that circulating PCSK9 is a biomarker of subclinical atherosclerotic disease.

## Figures and Tables

**Table 1:** Baseline characteristics of the IMPROVE study cohort stratified by quintiles of plasma PCSK9 levels (ng/mL).

PCSK9 (ng/mL)	1st Quintile (*n* = 734) 183 (155; 203)	2nd Quintile (*n* = 735) 251 (238; 264)	3rd Quintile (*n* = 735) 300 (287; 312)	4th Quintile (*n* = 735) 356 (340; 373)	5th Quintile (*n* = 734) 449 (416; 503)	*p* Value
Male, *n* (%)	482 (65.7)	381 (51.8)	347 (47.2)	277 (37.7)	271 (36.9)	<0.0001
Age (years)	64.7 ± 5.3	64.4 ± 5.1	64.4 ± 5.5	63.7 ± 5.4	63.7 ± 5.8	<0.0001
Diastolic blood pressure (mmHg)	84.1 ± 10.0	82.6 ± 9.9	81.5 ± 9.6	81.1 ± 9.8	80.7 ± 9.2	<0.0001
Systolic blood pressure (mmHg)	146.2 ± 19.1	142.6 ± 18.9	141.7 ± 17.8	140.8 ± 18.2	138.4 ± 17.7	<0.0001
Body mass index (Kg/m^2^)	27.6 ± 4.2	27.4 ± 4.4	27.3 ± 4.3	27.2 ± 4.4	26.9 ± 4.0	0.0004
Waist/hip ratio	0.94 ± 0.10	0.92 ± 0.10	0.92 ± 0.10	0.91 ± 0.10	0.91 ± 0.10	<0.0001
PH of hypercholesterolemia, *n* (%)	398 (54.2)	491 (67.0)	513 (69.9)	577 (78.6)	656 (89.4)	<0.0001
PH of hypertriglyceridemia, *n* (%)	125 (17.0)	149 (20.3)	200 (27.2)	196 (26.7)	279 (38.0)	<0.0001
PH of low HDL-cholesterol, *n* (%)	144 (19.6)	102 (13.9)	93 (12.7)	62 (8.4)	84 (11.4)	<0.0001
PH of hypertension, *n* (%)	577 (78.6)	497 (67.8)	514 (69.9)	484 (65.9)	464 (63.2)	<0.0001
PH of diabetes, *n* (%)	227 (30.9)	174 (23.7)	200 (27.2)	156 (21.2)	146 (19.9)	<0.0001
Educational level (study years)	10.8 ± 3.8	10.5 ± 3.9	10.3 ± 3.8	10.4 ± 3.9	10.3 ± 4.1	0.03
Total cholesterol (mg/dL)	203.9 ± 40.8	211.9 ± 43.5	213.7 ± 44.0	214.7 ± 44.8	215.5 ± 44.1	<0.0001
LDL-cholesterol (mg/dL)	132.4 ± 36.0	137.6 ± 38.2	138.0 ± 39.2	137.3 ± 41.0	137.3 ± 39.6	0.04
HDL-cholesterol (mg/dL)	47.0 ± 14.1	48.5 ± 14.2	48.6 ± 13.5	49.9 ± 13.5	50.1 ± 14.4	<0.0001
Triglycerides (mg/dL)	104 (77; 156)	110 (78; 159)	120 (84; 168)	117 (84; 168)	122 (86; 177)	0.0003
Uric acid (mg/dL)	5.5 (4.7; 6.3)	5.3 (4.5; 6.1)	5.2 (4.4; 6.0)	5.1 (4.3; 5.9)	5.0 (4.2; 5.8)	<0.0001
Blood glucose (mmol/L)	6.23 ± 1.6	5.97 ± 1.6	5.93 ± 1.8	5.69 ± 1.4	5.76 ± 1.7	<0.0001
Creatinine (μmol/L)	84.1 (73.8; 96.4)	79.4 (68.8; 90.4)	78.0 (68.4; 89.4)	76.5 (65.6; 87.2)	76.8 (66.9; 88.9)	<0.0001
Estimated GFR (mL/min)	84.3 ± 22.8	86.0 ± 23.3	83.9 ± 22.4	83.9 ± 22.5	80.2 ± 21.7	<0.0001

PCSK9, proprotein convertase subtilisin/kexin type 9; PH, personal history; LDL, low-density lipoproteins; HDL, high-density lipoproteins; GFR, glomerular filtration rate. Data are *n* (percentage) or mean ± SD, except for PCSK9, triglycerides, uric acid, and creatinine, which are summarized as median (1st and 3rd quartiles). Group differences were assessed by Student’s *t*-test for the numerical variables, by χ^2^-test for the categorical ones, and by Kruskal-Wallis for triglycerides, uric acid, and creatinine. The *p* values refer to the trends across PCSK9 quintiles. *p* values < 0.05 were considered statistically significant.

**Table 2:** Ultrasonographic variables of the IMPROVE study cohort stratified by quintiles of plasma PCSK9 levels (ng/mL).

PCSK9 (ng/mL)	1st Quintile (*n* = 734) 183 (155; 203)	2nd Quintile (*n* = 735) 251 (238; 264)	3rd Quintile (*n* = 735) 300 (287; 312)	4th Quintile (*n* = 735) 356 (340; 373)	5th Quintile (*n* = 734) 449 (416; 503)	*p* Value
IMTmean (mm)	0.89 (0.78; 1.02)	0.85 (0.75; 1.01)	0.86 (0.74; 0.99)	0.82 (0.73; 0.97)	0.81 (0.72; 0.96)	<0.0001
IMTmax (mm)	2.03 (1.55; 2.68)	1.85 (1.45; 2.50)	1.85 (1.45; 2.50)	1.76 (1.35; 2.48)	1.76 (1.30; 2.41)	<0.0001
IMTmean-max (mm)	1.43 (1.19; 1.69)	1.35 (1.14; 1.67)	1.36 (1.13; 1.63)	1.3 (1.09; 1.60)	1.27 (1.07; 1.60)	<0.0001
PF CC-IMTmean (mm)	0.72 (0.66; 0.78)	0.71 (0.65; 0.77)	0.7 (0.65; 0.76)	0.69 (0.64; 0.75)	0.69 (0.63; 0.74)	<0.0001
Fastest-IMTmax-progr (mm/y)	0.22 (0.11; 0.37)	0.22 (0.11; 0.34)	0.18 (0.11; 0.32)	0.17 (0.10; 0.31)	0.18 (0.09; 0.36)	0.01
ICCAD (mm)	8.04 (7.48; 8.60)	7.81 (7.21; 8.36)	7.76 (7.27; 8.33)	7.59 (7.11; 8.18)	7.50 (7.12; 8.03)	<0.0001
GSM of PF CC-IMTmean	41.2 (35.1; 50.4)	43.0 (36.0; 51.6)	44.3 (36.8; 54.0)	44.0 (36.4; 53.1)	46.1 (38.1; 54.6)	<0.0001
GSM of carotid plaque	29.8 (24.3; 36.6)	30.6 (24.9; 37.6)	31.7 (25.3; 39.6)	31.4 (25.1; 40.0)	32.6 (26.3; 39.7)	<0.0001

PCSK9, proprotein convertase subtilisin/kexin type 9; IMTmean, average of mean of intima-media thickness in left and right carotid arteries; IMTmax, highest value of maximum of intima-media thickness in left and right carotid arteries; IMTmean-max, mean of maximum intima-media thickness in left and right carotid arteries; PF CC-IMTmean, IMTmean measured in the 2nd cm of common carotids in plaque-free areas; Fastest-IMTmax-progr, the 15th month progression of IMTmax detected in the whole carotid tree regardless of location; ICCAD, average of the inter-adventitia diameter measurements carried out in plaque-free areas of the 2nd cm of left and right common carotid arteries; GSM, grey scale median of pixel distribution of the region of interest (cIMT or plaque). Data are expressed as median (1st and 3rd quartiles). Group differences were assessed by Kruskal-Wallis. The *p* values refer to the trend across PCSK9 quintiles. *p* values < 0.05 were considered statistically significant.

**Table 3:** Multivariable relationships between plasma PCSK9 levels and carotid IMT phenotypes, ICCAD and echolucency (Grey Scale Median; GSM).

	Model 0	Model 1
	Standardized Beta (95% CI); *p* Value	Standardized Beta (95% CI); *p* Value
IMTmean (mm)	−0.022 (−0.029, −0.016); <0.0001	0.029 (−0.005, 0.064); 0.09
IMTmax (mm)	−0.091 (−0.124, −0.059); <0.0001	0.015 (−0.020, 0.051); 0.38
IMTmean-max (mm)	−0.111 (−0.144, −0.078); <0.0001	0.022 (−0.013, 0.057); 0.21
PF CC-IMTmean (mm)	−0.135 (−0.168, −0.102); <0.0001	0.016 (−0.019, 0.051); 0.36
Fastest-IMTmax-progr (mm/y)	−0.025 (−0.059, 0.009); 0.14	0.007 (−0.002, 0.017); 0.13
ICCAD (mm)	−0.184 (−0.217, −0.152); <0.0001	−0.022 (−0.054, 0.011); 0.19
GSM of PF CC-IMTmean	0.094 (0.061, 0.127); <0.0001	0.032 (−0.005, 0.069); 0.08
GSM of carotid plaque	0.078 (0.045, 0.110); <0.0001	0.016 (−0.021, 0.053); 0.40

Acronyms as in the legend of Table 2. Standardized Beta values indicate change in the specific carotid phenotype associated with a standard deviation increment of plasma PCSK9 levels. Model 0: unadjusted; Model 1: adjusted for age, sex, latitude, and pharmacological treatment (i.e., therapy with statins, or fibrates, or with both statins and fibrates). *p* values < 0.05 were considered statistically significant.

## Data Availability

The data presented in this study are available on request from the corresponding author. The data are not publicly available due to ethical reasons.

## Supplementary Materials

The following are available online at https://www.mdpi.com/article/10.3390/biomedicines9070841/s1, Table S1: Baseline characteristics of the IMPROVE study cohort stratified by quintiles of plasma PCSK9 levels (ng/mL), Figure S1: Multivariable relationships between plasma PCSK9 levels and carotid IMT phenotypes, ICCAD and echolucency (Grey Scale Median; GSM) after stratification by sex, Figure S2: Multivariable relationships between plasma PCSK9 levels and carotid IMT phenotypes, ICCAD and echolucency (Grey Scale Median; GSM) after stratification by latitude, Figure S3: Multivariable relationships between plasma PCSK9 levels and carotid IMT phenotypes, ICCAD and echolucency (Grey Scale Median; GSM) after stratification by pharmacological treatment.
