# Supplementary material for: Relationship between Circulating PCSK9 and Markers of Subclinical Atherosclerosis—The IMPROVE Study
Source: Biomedicines. 2021 Jul 19;9(7):841. doi: 10.3390/biomedicines9070841 (PMC8301759; doi:10.3390/biomedicines9070841)
Supplement: Supplementary file 1 [file biomedicines-09-00841-s001.zip › biomedicines-1262361-supplementary.pdf]

Supplementary Data

RELATIONSHIP BETWEEN CIRCULATING PCSK9 AND MARKERS OF SUBCLINICAL ATHEROSCLEROSIS – THE IMPROVE STUDY

Daniela Coggi,[1] Beatrice Frigerio,[1] Alice Bonomi,[1] Massimiliano Ruscica,[2] Nicola Ferri,[3] Daniela Sansaro,[1] Alessio Ravani,[1] Palma Ferrante,[1] Manuela Damigella,[1] Fabrizio Veglia,[1] Nicolò Capra,[1] Maria G. Lupo,[3] Chiara Macchi,[2] Kai Savonen,[4,5] Angela Silveira,[6,7] Sudhir Kurl,[8] Philippe Giral,[9] Matteo Pirro,[10] Rona J. Strawbridge,[6,11,12] Bruna Gigante,[6] Andries J. Smit,[13] Elena Tremoli,[14] Mauro Amato,[1] and Damiano Baldassarre,[1,15,*] on behalf of the IMPROVE Study Group[§]

[1] Centro Cardiologico Monzino, IRCCS, Milan, Italy.

[2] Dipartimento di Scienze Farmacologiche e Biomolecolari, Università degli Studi di Milano, Milan, Italy.

[3] Dipartimento di Scienze del Farmaco, Università degli Studi di Padova, Padua, Italy.

[4] Foundation for Research in Health Exercise and Nutrition, Kuopio Research Institute of Exercise Medicine, Kuopio, Finland.

[5] Department of Clinical Physiology and Nuclear Medicine, Kuopio University Hospital, Kuopio, Finland.

[6] Division of Cardiovascular Medicine, Department of Medicine Solna, Karolinska Institutet, Stockholm, Sweden.

[7] Karolinska University Hospital, Solna, Stockholm, Sweden.

[8] Institute of Public Health and Clinical Nutrition, University of Eastern Finland, Kuopio Campus.

[9] Assistance Publique - Hopitaux de Paris; Service Endocrinologie-Metabolisme, Groupe Hôspitalier Pitié-Salpetriere, Unités de Prévention Cardiovasculaire, Paris, France.

[10] Internal Medicine, Angiology and Arteriosclerosis Diseases, Department of Medicine and Surgery, University of Perugia, Perugia, Italy.

[11] Institute of Health and Wellbeing, University of Glasgow, Glasgow, United Kingdom.

[12] Health Data Research UK.

[13] Department of Medicine, University Medical Center Groningen, Groningen & Isala Clinics Zwolle, Department of Medicine; the Netherlands.

[14] Maria Cecilia Hospital, Cotignola (RA), Italy.

[15] Department of Medical Biotechnology and Translational Medicine, Università degli Studi di Milano, Milan, Italy.

***Corresponding author:**

Damiano Baldassarre, PhD.

Address 1: Department of Medical Biotechnology and Translational Medicine, Università degli Studi di Milano, via Vanvitelli 32, 20129 - Milan, Italy.

Address 2: Centro Cardiologico Monzino, IRCCS, via Parea 4, 20138 - Milan, Italy.

Phone: +39-02-58002253

Fax: +39-02-58002623

E-mail 1: damiano.baldassarre@unimi.it

E-mail 2: damiano.baldassarre@ccfm.it

[§]The IMPROVE Study Group

Centro Cardiologico Monzino, IRCCS, Milan, Italy: Daniela Coggi, Beatrice Frigerio, Daniela Sansaro, Alessio Ravani, Palma Ferrante, Manuela Damigella, Fabrizio Veglia, Alice Bonomi, Nicolò Capra, Calogero Tedesco, Mauro Amato, Damiano Baldassarre.

Department of Medical Biotechnology and Translational Medicine, Università degli Studi di Milano, Milan, Italy: Damiano Baldassarre.

Maria Cecilia Hospital, Cotignola (RA), Italy: Elena Tremoli

Dipartimento di Scienze Farmacologiche e Biomolecolari, Università degli Studi di Milano, Milan, Italy: Laura Calabresi, Cesare R. Sirtori.

Division of Cardiovascular Medicine, Department of Medicine Solna, Karolinska Institutet, Stockholm, Sweden: Per Eriksson, Rona J. Strawbridge, Bruna Gigante, Angela Silveira, Anders Hamsten.

Karolinska University Hospital, Solna, Stockholm, Sweden: Angela Silveira, Per Eriksson, Anders Hamsten.

Institute of Health and Wellbeing, University of Glasgow, Glasgow, United Kingdom: Rona J. Strawbridge.

Health Data Research UK: Rona J. Strawbridge.

Division of Cardiovascular and Nutritional Epidemiology, Institute of Environmental Medicine, Karolinska Institutet: Karin Leander, Federica Laguzzi, Ulf de Faire

Cardiovascular Genetics, Institute Cardiovascular Science, University College of London, Rayne Building, University Street, London, United Kingdom: Steve E. Humphries, Jackie A. Cooper, Jayshree Acharya.

Foundation for Research in Health Exercise and Nutrition, Kuopio Research Institute of Exercise Medicine, Kuopio, Finland: Kai Savonen, Kirsi Huttunen, Eva Rauramaa, Ilkka M. Penttila, Jukka Törrönen.

Department of Clinical Physiology and Nuclear Medicine, Kuopio University Hospital, Kuopio, Finland: Kai Savonen.

Department of Medicine, University Medical Center Groningen, Groningen & Isala Clinics Zwolle, Department of Medicine; the Netherlands: Andries J. Smit, A.I. van Gessel, A.M van Roon, A. Nicolai, D.J. Mulder, G.H. Smeets.

Assistance Publique - Hopitaux de Paris; Service Endocrinologie-Metabolisme, Groupe Hôspitalier Pitié-Salpetriere, Unités de Prévention Cardiovasculaire, Paris, France: Philippe Giral, Anatole Kontush, Alain Carrié, Antonio Gallo.

Institute of Public Health and Clinical Nutrition, University of Eastern Finland, Kuopio Campus: Sudhir Kurl, J. Karppi, T. Nurmi, K. Nyyssönen, T.P. Tuomainen, J.Tuomainen, J. Kauhanen.

Internal Medicine, Angiology and Arteriosclerosis Diseases, Department of Medicine and Sugery, University of Perugia, Perugia, Italy: Matteo Pirro, M.R. Mannarino, G. Vaudo, V. Bianconi, E. Marini, F. Figorilli.

Supplementary Table S1. Baseline characteristics of the IMPROVE study cohort stratified by quintiles of plasma PCSK9 levels (ng/mL).

PCSK9 (ng/mL)	**1st quintile (n=734)** 183 (155; 203)	**2nd quintile (n=735)** 251 (238; 264)	**3rd quintile (n=735)** 300 (287; 312)	**4th quintile (n=735)** 356 (340; 373)	**5th quintile (n=734)** 449 (416; 503)	**P value**
**Latitude**						
Perugia, n (%)	25 (3.4)	70 (9.5)	114 (15.5)	159 (21.6)	173 (23.6)	
Milan, n (%)	82 (11.2)	85 (11.6)	91 (12.4)	89 (12.1)	205 (27.9)	
Paris, n (%)	35 (4.8)	91 (12.4)	109 (14.8)	136 (18.5)	125 (17.0)	**<0.0001**
Groningen, n (%)	51 (6.9)	133 (18.1)	147 (20.0)	109 (14.8)	75 (10.2)	
Stockholm, n (%)	107 (14.6)	134 (18.2)	119 (16.2)	111 (15.1)	60 (8.2)	
Kuopio, n (%)	434 (59.1)	222 (30.2)	155 (21.1)	131 (17.8)	96 (13.1)	
**Other anthropometric variables**						
Height (m)	1.70 ± 0.10	1.69 ± 0.10	1.67 ± 0.10	1.66 ± 0.10	1.65 ± 0.10	**<0.0001**
Weight (Kg)	80.2 ± 14.6	78.5 ± 15.6	76.5 ± 15.0	74.9 ± 15.4	73.3 ± 14.4	**<0.0001**
Waist (mm)	96.1 ± 12.5	94.7 ± 12.7	94.4 ± 12.5	92.9 ± 12.7	92.1 ± 12.0	**<0.0001**
Hip (mm)	102.4 ± 8.4	103.0 ± 9.7	102.7 ± 10.2	102.4 ± 10.0	101.2 ± 9.4	**0.01**
**Smoking habits**						
Current smokers, n (%)	117 (15.9)	110 (15.0)	95 (12.9)	108 (14.7)	114 (15.5)	
Former smokers, n (%)	272 (37.1)	290 (39.5)	274 (37.3)	261 (35.5)	263 (35.8)	0.57
Never smokers, n (%)	345 (47.0)	335 (45.6)	366 (49.8)	366 (49.8)	357 (48.6)	

Pack-years	0.7 (0.0; 18.0)	1.5 (0.0; 18.5)	0.0 (0.0; 19.0)	0.0 (0.0; 18.0)	0.6 (0.0; 17.3)	0.57
**Physical activity**						
Low, n (%)	91 (12.4)	119 (16.2)	154 (21.1)	159 (21.6)	205 (28.0)	
Medium, n (%)	276 (37.6)	352 (48.0)	345 (47.3)	335 (45.6)	320 (43.7)	**<0.0001**
High, n (%)	367 (50.0)	262 (35.7)	231 (31.6)	241 (32.8)	207 (28.3)	
**Family history of**						
Coronary Heart Disease, n (%)	501 (71.1)	469 (67.5)	456 (64.0)	448 (62.4)	418 (58.6)	**<0.0001**
CerebroVascular Disease, n (%)	277 (37.7)	263 (35.8)	263 (35.8)	250 (34.0)	262 (35.7)	0.69
Peripheral Vascular Disease, n (%)	99 (13.5)	91 (12.4)	64 (8.7)	91 (12.4)	94 (12.8)	**0.045**
Hyperlipidemia, n (%)	237 (32.3)	279 (38.0)	311 (42.3)	335 (45.6)	401 (54.6)	**<0.0001**
Hypertension, n (%)	459 (62.5)	405 (55.1)	420 (57.1)	426 (58.0)	449 (61.2)	**0.03**
Diabetes, n (%)	301 (41.0)	260 (35.4)	253 (34.4)	270 (36.7)	221 (30.1)	**0.001**
**Other biochemical variables**						
Leucocytes (WBC) (x 10^9/L)	6.02 ± 1.6	6.16 ± 1.7	6.12 ± 1.6	6.19 ± 1.6	6.21 ± 2.3	0.053
Neutrophils (%)	56.85 ± 9.7	58.02 ± 9.1	57.21 ± 8.9	57.75 ± 9.0	55.98 ± 8.8	0.058
Lymphocytes (%)	33.57 ± 8.6	32.28 ± 8.1	33.00 ± 8.2	32.67 ± 8.2	33.95 ± 8.4	0.24
Monocytes (%)	6.82 ± 2.2	6.74 ± 2.2	6.67 ± 2.1	6.49 ± 2.1	7.00 ± 2.2	0.50
Eosinophils (%)	3.16 ± 2.2	3.03 ± 2.0	3.06 ± 2.1	2.94 ± 1.9	2.96 ± 1.9	0.13
Basophils (%)	0.4 (0.0; 0.7)	0.4 (0.0; 0.7)	0.5 (0.2; 0.8)	0.5 (0.2; 0.7)	0.5 (0.3; 0.8)	**0.02**

Erythrocytes (RBC) (x 10^{12}/L)	4.69 ± 0.4	4.66 ± 0.4	4.65 ± 0.4	4.65 ± 0.4	4.63 ± 0.4	**0.01**
Haemoglobin (g/dL)	14.45 ± 1.2	14.26 ± 1.2	14.14 ± 1.1	14.11 ± 1.1	13.99 ± 1.1	**<0.0001**
Haematocrit (%)	42.58 ± 3.4	42.19 ± 3.7	42.18 ± 4.4	42.12 ± 3.6	41.95 ± 3.2	**0.002**
MCV (fl)	90.98 ± 4.5	90.44 ± 4.5	90.51 ± 5.2	90.75 ± 5.1	90.73 ± 4.4	0.74
MCH (pg)	30.92 ± 1.8	30.64 ± 1.7	30.54 ± 1.7	30.48 ± 1.7	30.28 ± 1.7	**<0.0001**
MCHC (g/dL)	33.98 ± 0.9	33.88 ± 1.1	33.69 ± 1.1	33.54 ± 1.1	33.36 ± 1.0	**<0.0001**
Platelets (x 10^{9}/L)	225.5 ± 56.0	236.4 ± 56.9	237.6 ± 57.2	241.1 ± 58.1	246.5 ± 55.0	**<0.0001**
hs-CRP (mg/L)	1.7 (0.7; 3.4)	2.1 (0.8; 3.7)	1.9 (0.7; 3.8)	2.0 (0.8; 3.8)	1.8 (0.8; 3.3)	0.60
**Food items**						
Wine, n (%)	278 (7.59)	273 (7.45)	295 (8.05)	287 (7.83)	363 (9.91)	**<0.0001**
Beer, n (%)	177 (4.83)	140 (3.82)	124 (3.38)	110 (3)	93 (2.54)	**<0.0001**
Spirits, n (%)	158 (4.31)	124 (3.38)	96 (2.62)	90 (2.46)	72 (1.96)	**<0.0001**
Fruit, n (%)	679 (18.51)	679 (18.51)	699 (19.06)	695 (18.95)	698 (19.03)	0.06
Milk, n (%)	590 (16.12)	566 (15.46)	580 (15.84)	553 (15.11)	550 (15.02)	0.06
Coffee, n (%)	663 (18.1)	664 (18.13)	658 (17.96)	661 (18.05)	656 (17.91)	0.98
Tea, n (%)	326 (8.9)	345 (9.42)	271 (7.4)	267 (7.29)	236 (6.44)	**<0.0001**
Meat, n (%)	719 (19.65)	716 (19.57)	722 (19.73)	721 (19.7)	720 (19.68)	0.50
Fish, n (%)	669 (18.28)	655 (17.9)	658 (17.98)	674 (18.42)	674 (18.42)	0.19
Eggs, n (%)	619 (16.92)	577 (15.77)	553 (15.11)	571 (15.61)	515 (14.07)	**<0.0001**

**Pharmacological therapies**						
Statins, n (%)	169 (23)	234 (32)	279 (38)	369 (50)	423 (58)	**<0.0001**
Fibrates, n (%)	19 (2.6)	25 (3.4)	50 (6.8)	53 (7.2)	136 (19)	**<0.0001**
Fish oil, n (%)	24 (3.3)	20 (2.7)	20 (2.7)	21 (2.9)	40 (5.4)	**0.02**
Other lipid-lowering drug, n (%)	3 (0.4)	9 (1.2)	2 (0.3)	4 (0.5)	5 (0.7)	0.23
Beta-blockers, n (%)	194 (26)	165 (22)	183 (25)	168 (23)	162 (22)	0.23
Calcium antagonists, n (%)	122 (17)	111 (15)	136 (19)	109 (15)	119 (16)	0.33
ACE inhibitors, n (%)	160 (22)	128 (17)	131 (18)	154 (21)	145 (20)	0.15
ARB, n (%)	134 (18)	98 (13)	110 (15)	110 (15)	108 (15)	0.11
Diuretics, n (%)	160 (22)	159 (22)	168 (23)	181 (25)	184 (25)	0.39
Anti-platelet agents, n (%)	150 (20)	106 (14)	114 (16)	110 (15)	134 (18)	**0.01**
Insulin, n (%)	40 (5.4)	33 (4.5)	26 (3.5)	17 (2.3)	24 (3.3)	**0.02**
Estrogen supplement, n (%)	52 (7.1)	47 (6.4)	46 (6.3)	52 (7.1)	29 (4.0)	0.08

PCSK9, proprotein convertase subtilisin/kexin type 9; WBC, white blood cells; RBC, red blood cells; MCV, mean corpuscular volume; MCH, mean corpuscular haemoglobin; MCHC, mean corpuscular haemoglobin concentration; hs-CRP, high-sensitivity C-reactive protein; ACE, angiotensin-converting enzyme; ARB, angiotensin-2 receptor blockers. Data are n (percentage) or mean ± SD, except for PCSK9, pack-years, basophils, and hs-CRP, which are summarized as median (1st and 3rd quartiles). Group differences were assessed by Student's t-test for the numerical variables, by χ2-test or Fisher for the categorical ones, and by Kruskal-Wallis for pack-years, basophils, and hs-CRP. Estrogen supplementation was calculated only in women. The P_{values} refer to the trends across PCSK9 quintiles. P_{values} <0.05 were considered statistically significant.

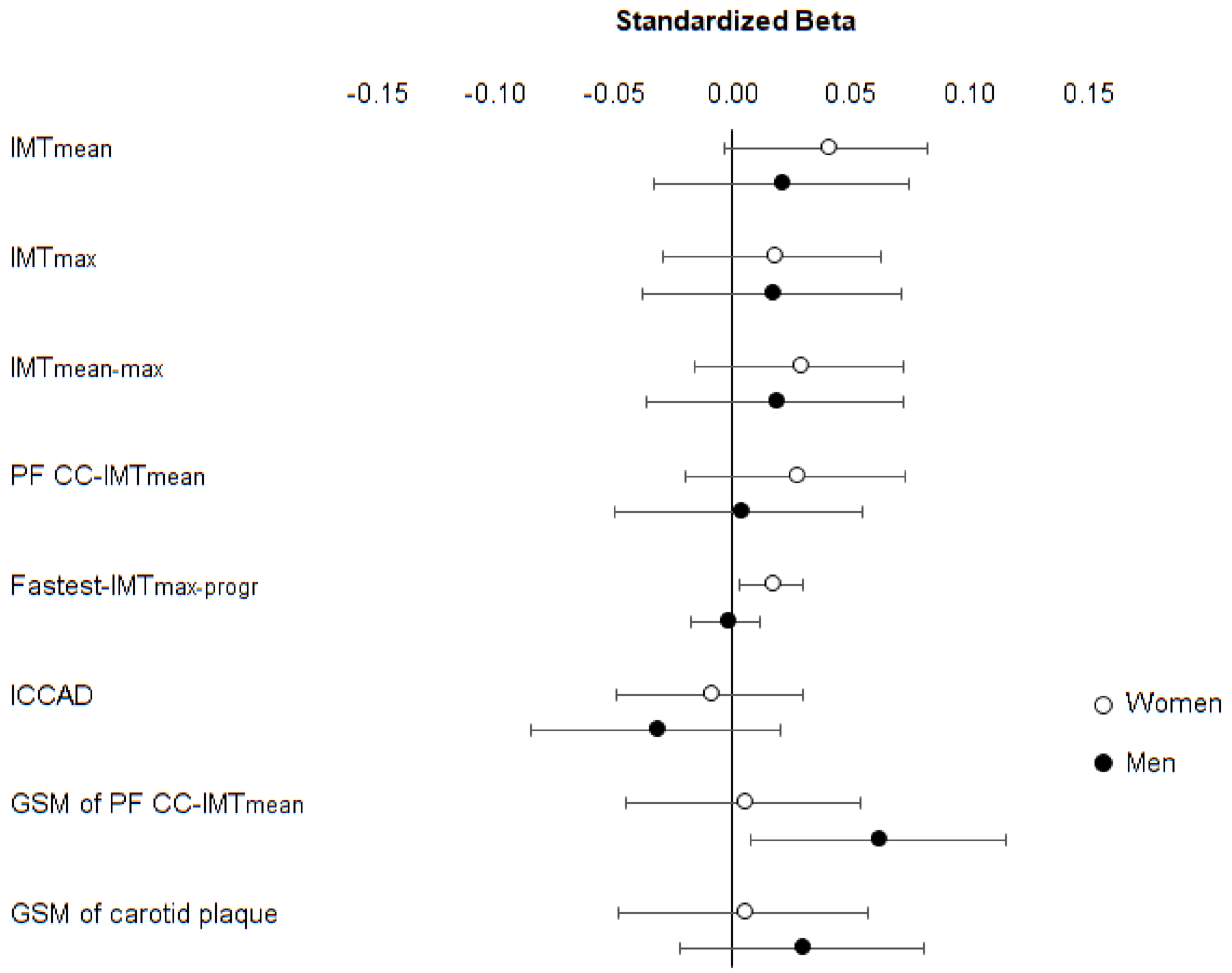

Supplementary Figure S1 Multivariable relationships between plasma PCSK9 levels and carotid IMT phenotypes, ICCAD and echolucency (Grey Scale Median; GSM) after stratification by sex.
PCSK9, proprotein convertase subtilisin/kexin type 9; IMT_{mean}, average of mean of intima-media thickness in left and right carotid arteries; IMT_{max}, highest value of maximum of intima-media thickness in left and right carotid arteries; $IMT_{mean-max}$, mean of maximum intima-media thickness in left and right carotid arteries; PF CC-IMT_{mean}, IMT_{mean} measured in the 2nd cm of common carotids in plaque-free areas; Fastest-$IMT_{max-progr}$, the 15-month progression of IMT_{max} detected in the whole carotid tree regardless of location; ICCAD, average of the inter-adventitia diameter measurements carried out in plaque-free areas of the 2nd cm of left and right common carotid arteries; GSM, grey scale median of pixels distribution of the region of interest (cIMT or plaque).

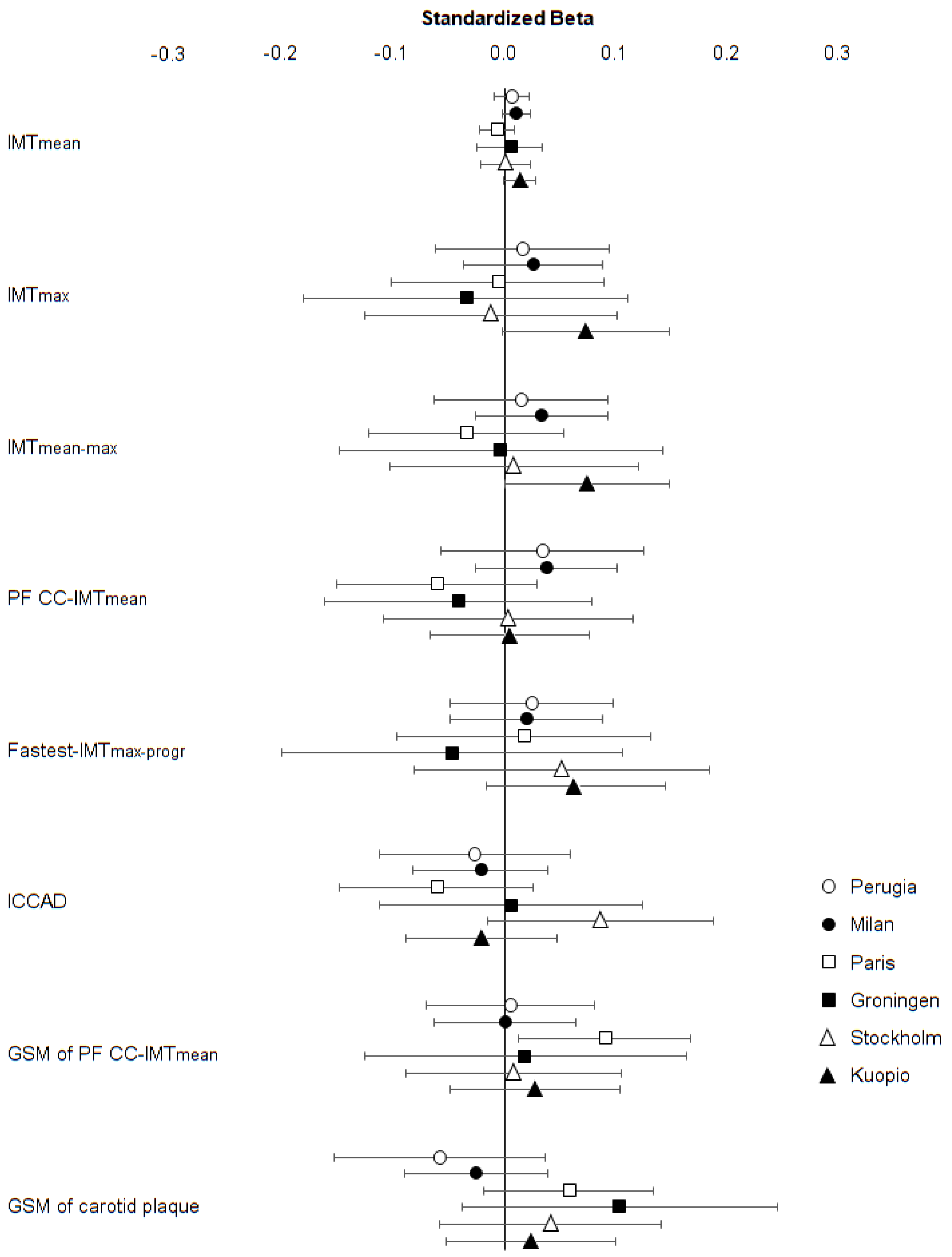

Supplementary Figure S2 Multivariable relationships between plasma PCSK9 levels and carotid IMT phenotypes, ICCAD and echolucency (Grey Scale Median; GSM) after stratification by latitude.

PCSK9, proprotein convertase subtilisin/kexin type 9; IMT_{mean}, average of mean of intima-media thickness in left and right carotid arteries; IMT_{max}, highest value of maximum of intima-media thickness in left and right carotid arteries; $IMT_{mean-max}$, mean of maximum intima-media thickness in left and right carotid arteries; PF CC-IMT_{mean}, IMT_{mean} measured in the 2nd cm of common carotids in plaque-free areas; Fastest-$IMT_{max-progr}$, the 15-month progression of IMT_{max} detected in the whole carotid tree regardless of location; ICCAD, average of the inter-adventitia diameter measurements carried out in plaque-free areas of the 2nd cm of left and right common carotid arteries; GSM, grey scale median of pixels distribution of the region of interest (cIMT or plaque).

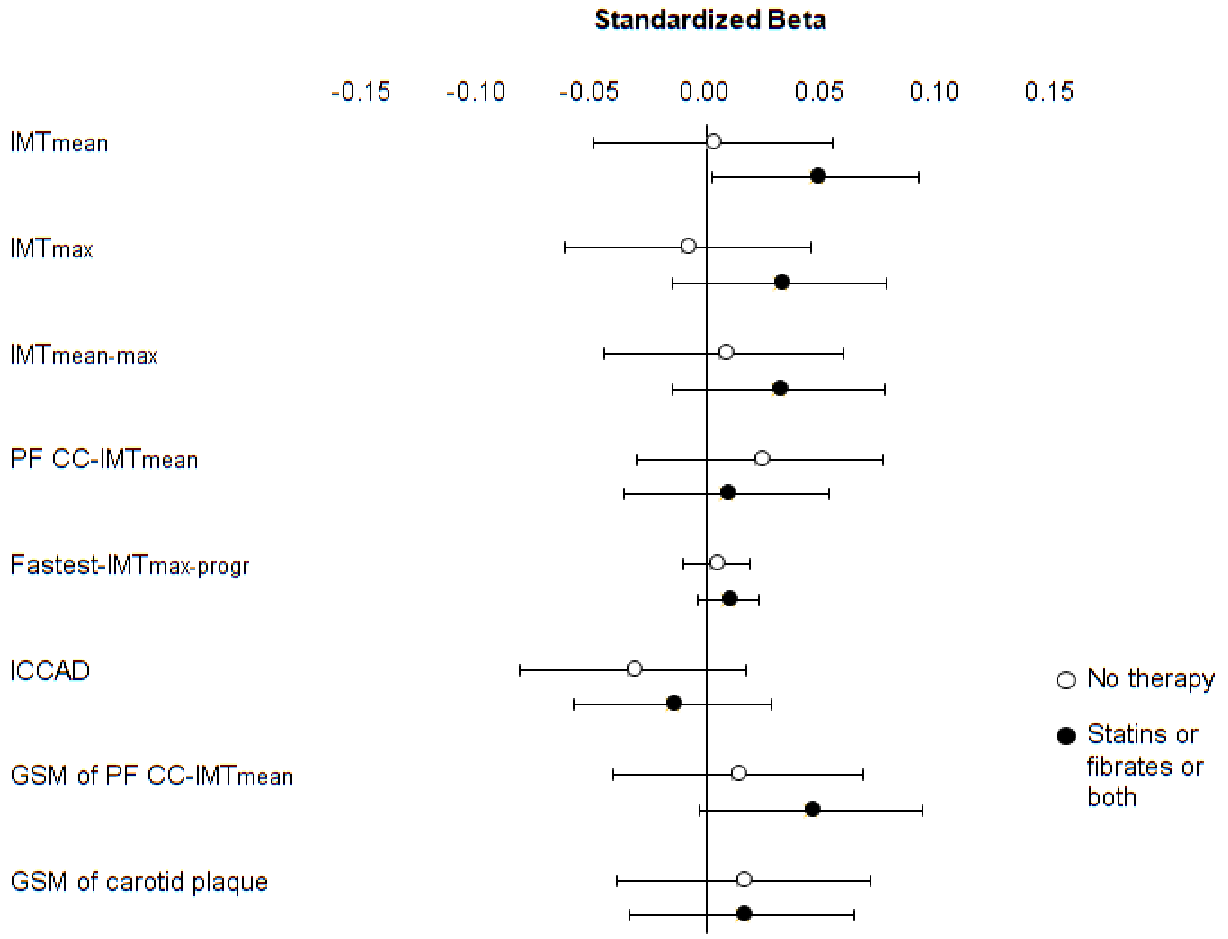

Supplementary Figure S3 Multivariable relationships between plasma PCSK9 levels and carotid IMT phenotypes, ICCAD and echolucency (Grey Scale Median; GSM) after stratification by pharmacological treatment.

PCSK9, proprotein convertase subtilisin/kexin type 9; IMT_{mean}, average of mean of intima-media thickness in left and right carotid arteries; IMT_{max}, highest value of maximum of intima-media thickness in left and right carotid arteries; $IMT_{mean-max}$, mean of maximum intima-media thickness in left and right carotid arteries; PF CC-IMT_{mean}, IMT_{mean} measured in the 2nd cm of common carotids in plaque-free areas; Fastest-$IMT_{max-progr}$, the 15-month progression of IMT_{max} detected in the whole carotid tree regardless of location; ICCAD, average of the inter-adventitia diameter measurements carried out in plaque-free areas of the 2nd cm of left and right common carotid arteries; GSM, grey scale median of pixels distribution of the region of interest (cIMT or plaque).
